# Integrated application of uniform design and least-squares support vector machines to transfection optimization

**DOI:** 10.1186/1472-6750-9-52

**Published:** 2009-05-31

**Authors:** Jin-Shui Pan, Mei-Zhu Hong, Qi-Feng Zhou, Jia-Yan Cai, Hua-Zhen Wang, Lin-Kai Luo, De-Qiang Yang, Jing Dong, Hua-Xiu Shi, Jian-Lin Ren

**Affiliations:** 1 Division of Gastroenterology, Zhongshan Hospital, Gastroenterology Institute of Xiamen University, Gastroenterology Center of Xiamen, Xiamen 361004, Fujian Province, PR China; 2 Division of Infectious Diseases, the 174th Hospital of PLA, Xiamen 361003, Fujian Province, PR China; 3 Department of Automation, Xiamen University, Xiamen 361005, Fujian Province, PR China

## Abstract

**Background:**

Transfection in mammalian cells based on liposome presents great challenge for biological professionals. To protect themselves from exogenous insults, mammalian cells tend to manifest poor transfection efficiency. In order to gain high efficiency, we have to optimize several conditions of transfection, such as amount of liposome, amount of plasmid, and cell density at transfection. However, this process may be time-consuming and energy-consuming. Fortunately, several mathematical methods, developed in the past decades, may facilitate the resolution of this issue. This study investigates the possibility of optimizing transfection efficiency by using a method referred to as least-squares support vector machine, which requires only a few experiments and maintains fairly high accuracy.

**Results:**

A protocol consists of 15 experiments was performed according to the principle of uniform design. In this protocol, amount of liposome, amount of plasmid, and the number of seeded cells 24 h before transfection were set as independent variables and transfection efficiency was set as dependent variable. A model was deduced from independent variables and their respective dependent variable. Another protocol made up by 10 experiments was performed to test the accuracy of the model. The model manifested a high accuracy. Compared to traditional method, the integrated application of uniform design and least-squares support vector machine greatly reduced the number of required experiments. What's more, higher transfection efficiency was achieved.

**Conclusion:**

The integrated application of uniform design and least-squares support vector machine is a simple technique for obtaining high transfection efficiency. Using this novel method, the number of required experiments would be greatly cut down while higher efficiency would be gained. Least-squares support vector machine may be applicable to many other problems that need to be optimized.

## Background

Central to life functions, protein expression in normal and diseased states is essential for quantifying altered patterns of gene expression. This is especially true in the era that the sequencing of human genome has been finished. To gain insights into protein expression, we have to transfect cells with kinds of expression vectors, based on plasmid, viral vector, or transposon, etc. Transfection may be one of the commonest but indispensable procedures for cellular biology. However, in the process of evolution, eukaryotic cells tend to have low transfection efficiency in order to protect their genomes from exogenous insults. Transfection difficulty manifests itself, especially in the cotransfection of mammalian cells. Theoretically, if the transfection efficiency of single kind of plasmid is E, which ranges from 0 to 1, the efficiency of double and triple cotransfection may decline to E^2 ^and E^3^, respectively. Therefore, it is of great importance to improve efficiency.

In order to enhance the transfection, several kinds of strategies are developed, which are categorized into two types: viral gene delivery carriers and non-viral gene delivery carriers. In non-viral gene delivery carriers, cationic liposomes has the widest application. Cationic liposomes are positively charged liposomes which interact with the negatively charged DNA molecules to form a stable complex. Cationic liposomes consist of a positively charged lipid and a co-lipid. A variety of positively charged lipid formulations are commercially available and many other are under development. Lipofection, one of the most frequently cited cationic lipids, was first reported by Felgner in 1987 to deliver genes to cells in culture [1]. Lipofection has been used to deliver linear DNA, plasmid DNA, and RNA to a variety of cells in culture. Liposomes offer several advantages in delivering genes to cells. (1) Liposomes have the ability to combine both with negatively and positively charged molecules. (2) Liposomes offer a degree of protection to the DNA from degradative processes. (3) Liposomes carry large pieces of DNA, potentially as large as a chromosome. (4) Liposomes can be targeted to specific cells or tissues. In addition, liposomes overcome problems inherent with viral vectors – specific concerns over immunogenicity and replication competent virus contamination. Liposomes resulted in a highly adaptable and flexible system capable of gene delivery both in vitro and in vivo. Current limitations regarding in vivo application of liposomes revolve around the low transfection efficiencies and transient gene expression. Also, liposomes display a small degree of cellular toxicity and appear to be inhibited by serum components. The ability to overcome these problems should greatly facilitate their application to a variety of gene delivery mechanisms.

Several factors have significant effects on the transfection efficiency of cationic liposomes, such as vigor of the host cells, the amount of plasmid, the amount of transfection agent, and the density of cells. However, it is hard to control vigor of host cells which has not a quantitative index. The other three factors are controllable in transfection, which can be adjusted according to the host cells and transfection agents. However, the adjustment of these three factors is a time and energy-consuming work. For most researchers, they may spend two to three months on optimizing transfection. Fortunately, several mathematical methods offer promising avenues to the resolution of this issue.

There are several ways to perform computer experiments, such as Latin Hypercube Sampling (LHS) and Uniform Design (UD). LHS was brought up by three scholars in the North American [2]. Uniform design, abbreviated as UD, was first developed by Fang *et al *in nineteen eighty [3]. UD seeks design points that are scattered uniformly on the domain. It has been popular since 1980. The main advantages of UD may be generalized as the following: first, it has the ability to greatly reduce the number of experiments while not to alter the representativeness; second, it generates a regression model based on the results and it's able to predict at what independent variables the dependent variable may gain the maximum.

As a relatively new algorithm used for classification and regression, support vector machine (SVM) was developed in the 1990s [4,5]. It is a desired method for estimation based on finite-sample and therefore is able to solve a lot of practical problems in case of limited samples. Their practical successes may be attributed to solid theoretical foundations based on Vapnik Chervonenkis theory, and to the minimization of structural risk [6]. In order to implement the SVM into our transfection optimization, the least squares support vector machines (LS-SVM) was used, which has a growing popularity for regression problems [7]. It can be argued that LS-SVM would yield better generalization for regression problems on finite samples [8].

## Results and discussion

**Figure 1:**
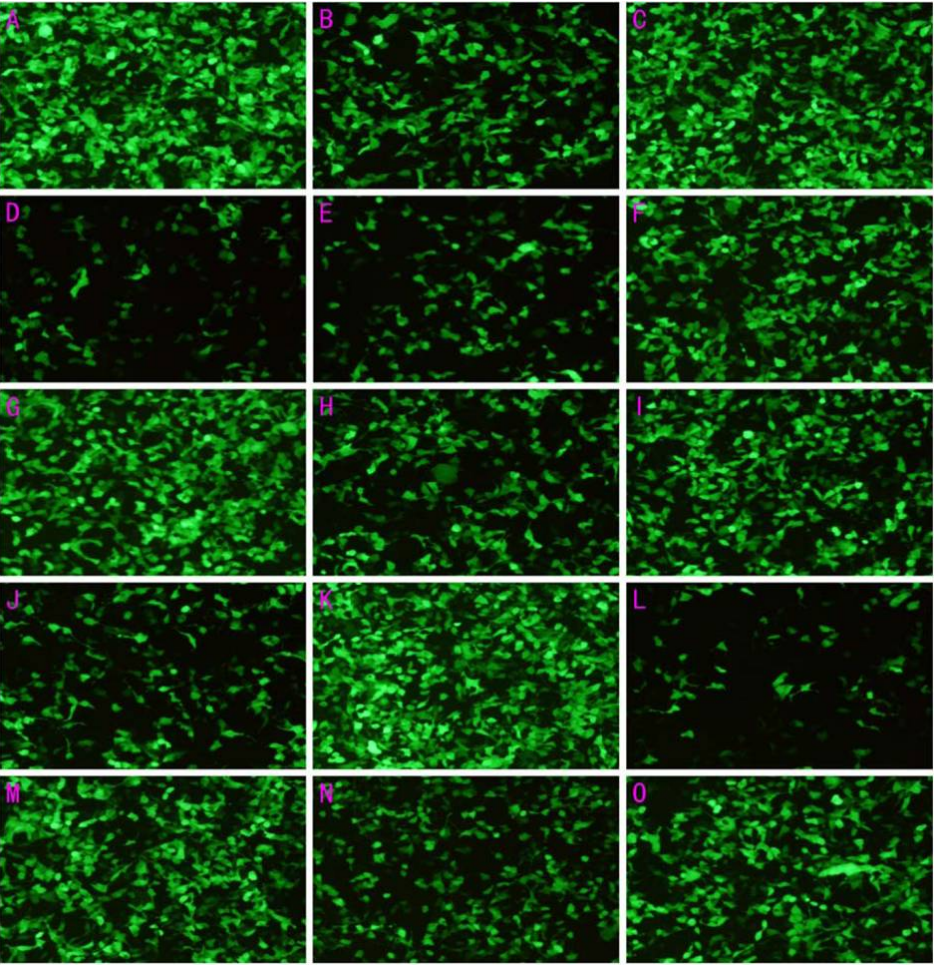
**Varied expression of green fluorescent protein under different transfection conditions**.

**Figure 2:**
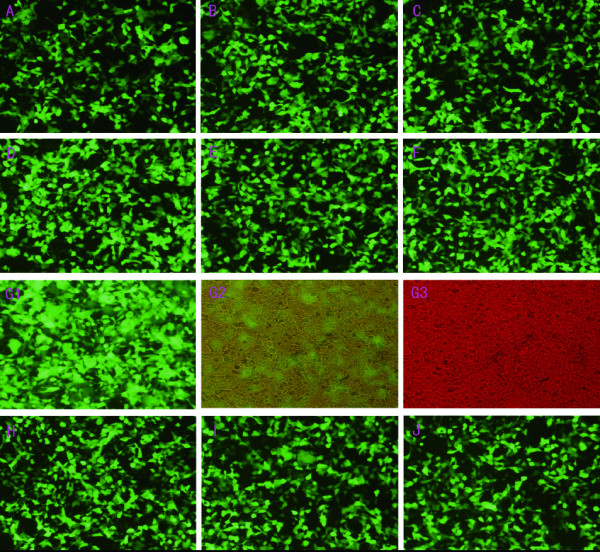
**Varied expression of green fluorescent protein under the conditions centering to the predicted optimal transfection conditions**. G1, G2, G3 showed the same visual field observed under the green, yellow and red light, respectively.

**Figure 3:**
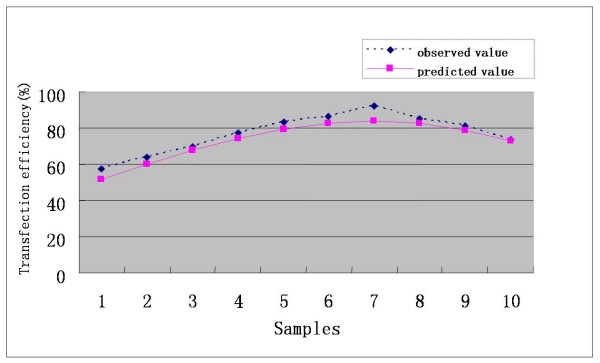
**Coincidence between observed values and predicted values based on LS-SVM**.

As was shown in Table 1 and Figure 1, transfection efficiency varied greatly with the changes of the amount of plasmid, LipofectAMINE, and the number of seeded cells. If these three independent varies did not match, transfection efficiency would decline sharply. In Table 1 and Figure 1, experiment L has the lowest efficiency (13.49%) for the ration of plasmid to transfection agent is too low, while experiment K has the highest efficiency owing to the designed ratios between the three independent factors. According to the established model, transfection efficiency would gain the maximum if 2.1×10^5 ^of cells, 0.66 *μ*g of plasmid and 1.32 *μ*g of LipofectAMINE were used. And this was accord with the observed data (Table 2 and Figure 2). More than that, there was a high degree of coincidence between calculated transfection efficiency and the deduced date from the model (Figure 3). Thus, by virtue of UD and LS-SVM, only 15 experiments, which can be performed in two 24-well plates, are needed to get the optimal transfection conditions whereas more than 15^3 ^experiments are needed to attain the expected purpose by using traditional method. What's more, if more accurate conditions were demanded, the number of experiments would greatly exceed 15^3^.

**Table 1:** Protocol of experiments for generating the model

Sequence Number	Number of Cells (× 105)*	Amount of plasmid (*μ*g) *	Amount of LipofectAMINE (*μ*L) *	Expression ratio of GFP %	Fitted Values %
A	11.0	100.50	80.88	64.74	64.50
B	21.1	50.25	40.56	45.58	45.72
C	31.2	140.70	111.12	68.37	68.06
D	41.3	20.10	131.28	19.60	20.25
E	51.4	120.60	10.32	30.61	31.04
F	61.5	70.35	60.72	56.16	56.09
G	71.6	80.40	151.44	75.24	74.80
H	81.7	40.20	101.04	48.78	48.85
I	91.8	150.75	50.64	59.19	59.06
J	101.9	30.15	20.40	38.23	38.51
K	112.0	110.55	141.36	82.53	81.94
L	122.1	10.05	70.80	13.49	14.26
M	132.2	130.65	90.96	71.67	71.29
N	142.3	90.45	30.48	51.96	51.97
O	152.4	60.30	121.20	61.85	61.67

*: In these independent variables, the numbers outside of round brackets refer to serial numbers of their levels, and the numbers inside of round brackets refer to the actual dosages.

**Table 2:** Protocol of experiments for testing accuracy of the model

Sequence Number	Number of Cells (× 10^5^)	Amount of plasmid (*μ*g)	Amount of LipofectAMINE (*μ*L)	Expression ratio of GFP (%)	Fitted Values (%)
A	1.50	0.30	0.60	57.25	50.07
B	1.60	0.36	0.72	63.56	58.98
C	1.70	0.42	0.84	69.73	67.57
D	1.80	0.48	0.96	77.64	75.13
E	1.90	0.54	1.08	83.47	80.97
F	2.00	0.60	1.20	86.42	84.47
G	2.10	0.66	1.32	92.32	85.12
H	2.20	0.72	1.44	85.19	82.59
I	2.30	0.78	1.56	81.29	76.73
J	2.40	0.84	1.68	73.61	67.62

The proper setting of LS-SVM model training parameters was tuned by grid search. The most common performance assessment method is probably the k-fold cross-validation [9] and the leave-one-out procedure. In the k-fold cross-validation, the training data are randomly split into k mutually exclusive subsets (the folds) with approximately equal sizes. The resulting LS-SVM model is obtained by training on k-1 subsets and then the model is tested on the remaining one subset. This procedure is repeated for k times and in this fashion each subset is used for testing only once. By averaging the test errors over the k trials it gives an estimate of the expected generalization error. The leave-one-out procedure can be viewed as an extreme form of the k-fold cross-validation with k equal to the number of examples. Leave-one-out is known as an unbiased estimation method for small-samples problems, such as our application. Therefore, fifteen times of training and test repeated on a pair of parameters and each MSE value for a pair of parameters were reported by the leave-one-out procedure. Part of results was listed in Table 3. As was shown in Table 3, the minimum MSE is found at a pair of parameters (*γ *= 42, C = 1500), and then the LS-SVM model obtains a peak estimated performance. After the optimal parameters for model construction are known, the according model (final model) is validated by predicting the validation data and comparing these predictions with the real observations.

**Table 3:** MSE of LS-SVM model based on training parameters tuning

C	...	1200	1300	1400	1500	1600	1700	...
*γ*								
...	...	...	...	...	...	...	...	...
32		6.4129	6.4572	6.4789	6.511	6.5446	6.5762	...
36		6.2583	6.2794	6.2943	6.3152	6.323	6.3628	...
40		6.2155	6.207	6.2024	6.2068	6.2047	6.2135	...
42		6.2271	6.2013	6.1901	**6.1810**	6.1815	6.1821	
48		6.4039	6.3349	6.2821	6.2379	6.2074	6.1829	
50		6.504	6.4205	6.3529	6.2984	6.254	6.2153	
60		7.2761	7.1095	6.9685	6.848	6.7444	6.6548	
								
...	...	...	...	...	...	...	...	...

**Figure 4:**
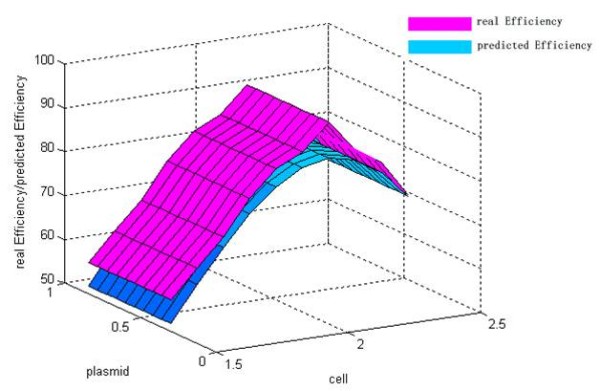
**Response surface showing the effect of plasmid and cell on transfection efficiency**. Pink response surface representing the real efficiency and blue response surface representing the predicted efficiency.

**Figure 5:**
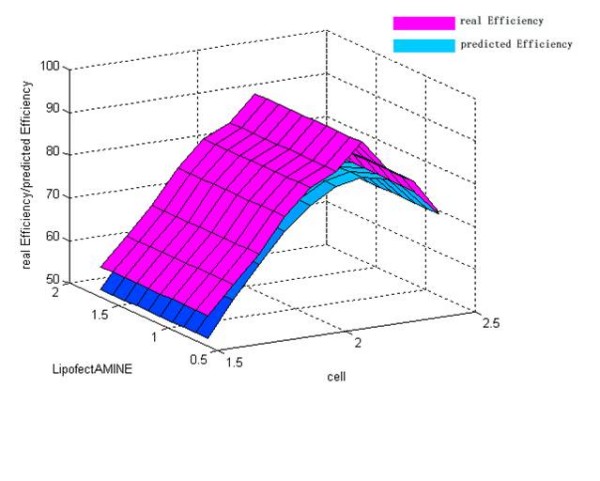
**Response surface showing the effect of LipofectAMINE and cell on transfection efficiency**. Pink response surface representing the real efficiency and blue response surface representing the predicted efficiency.

**Figure 6:**
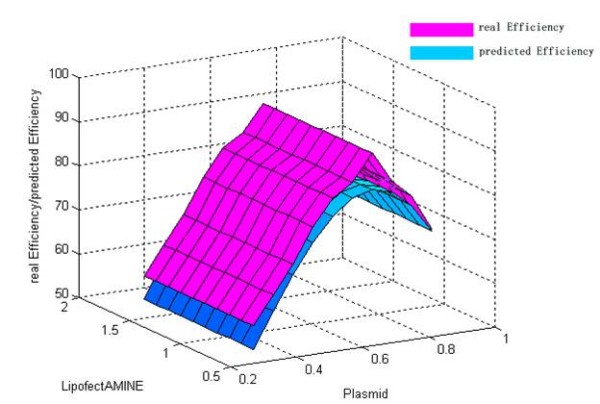
**Response surface showing the effect of LipofectAMINE and plasmid on transfection efficiency**. Pink response surface representing the real efficiency and blue response surface representing the predicted efficiency.

The discrepancy between the predicted value and their respective observed data was listed in Table 4. From Table 4, it could be find that the maximum of observed data was N7 (92.32%) and the maximum of predicted values based on LS-SVM was also N7 (84.04%). The error ratio between observed data and predicted value of LS-SVM was less than 10%. Thus, LS-SVM has an excellent predicted ability (generalization ability) on our problem. The mutual influence between the predicted value and two of all the three variables was shown in Figure 4, 5 and 6. In a three dimensional surface, each mesh point in the (x, y)-plane stood for a variable combination and the z-axis stood the predicted value. Figure 4, 5 and 6 showed that the change of LS-SVM predicted value on ten test samples was consistent with observed data.

**Table 4:** Coincidence between the observed data and the predicted values

	real observation	predicted value	Net difference	Error ratio
N1	57.25	51.91	5.34	0.093289
N2	63.56	59.86	3.70	0.058202
N3	69.73	67.43	2.30	0.033020
N4	77.64	74.10	3.54	0.045576
N5	83.47	79.38	4.09	0.048986
N6	86.42	82.81	3.61	0.041720
N7	92.32	84.04	8.28	0.089702
N8	85.19	82.81	2.38	0.027934
N9	81.29	79.03	2.26	0.027834
N10	73.61	72.74	0.87	0.011860

**Figure 7:**
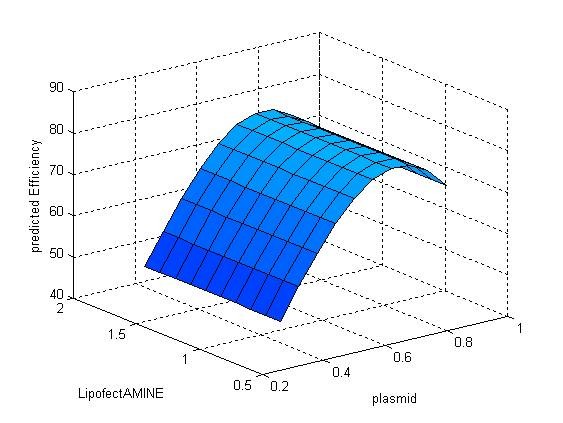
**Response surface showing the effect of random alteration of the seeded cells density on transfection efficiency**.

**Figure 8:**
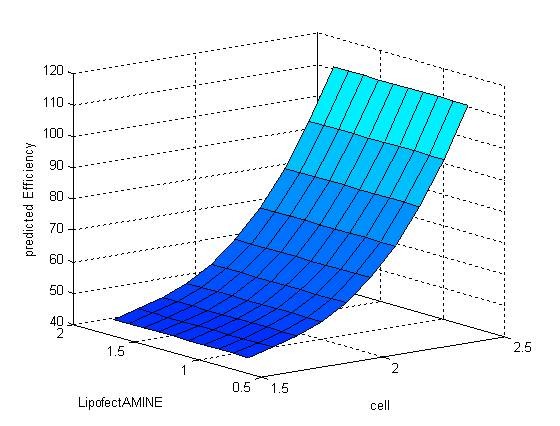
**Response surface showing the effect of random alteration of the amount of plasmid on transfection efficiency**.

**Figure 9:**
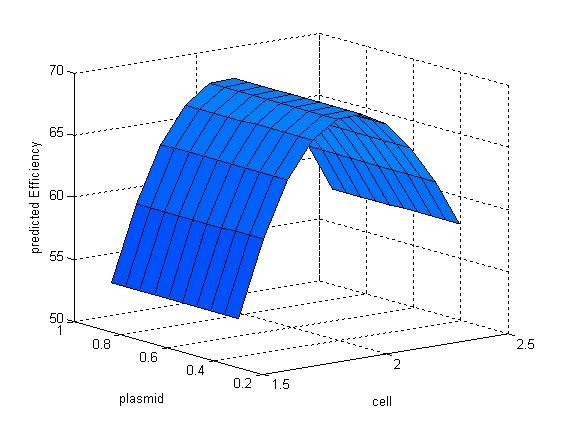
**Response surface showing the effect of random alteration of LipofectAMINE on transfection efficiency**.

The contribution of a specific independent variable was also evaluated. Table 5 showed the average MSE when one specific variable was ignored. As is indicated by Table 5 and Figure 7, 8 and 9, amount of plasmid has the most significant effect on transfection efficiency, followed by amount of LipofectAMINE, while the density of seeded cells has the least effect on transfection efficiency. And this result coincided with our experience.

**Table 5:** Contribution analysis of independent variables

variable	average predicated value										average MSE
	N1	N2	N3	N4	N5	N6	N7	N8	N9	N10	
x1	49.97	57.23	64.16	70.35	75.38	78.91	80.62	80.30	77.82	73.18	47.1928
x2	45.28	47.28	49.62	52.87	57.59	64.22	73.10	84.41	98.16	114.19	504.2573
x3	53.97	60.00	64.44	67.32	68.73	68.79	67.64	65.44	62.37	58.61	225.4837

x1: density of seeded cells; x2: amount of plasmid; x3: amount of LipofectAMINE

Owing to UD, the amount of test points required can be enormously reduced, especially when the experimental region has many factors and multiple levels, while the results that reflect the major characteristics of the experimental system are ensured. As an efficient fractional factorial design, UD has been widely applied in manufacturing, system engineering, pharmaceutics, and natural sciences in the past decades [10-12]. The UD was used in this research to describe factors that significantly influence transfection efficiency to obtain a smaller, more manageable set. To perform a computer experiment, in order to have a wide coverage of the entire design region with a limited number of runs, UD is a good recommendation.

The SVM is a machine learning technique with a strong theoretical foundation that has been used to improve classification accuracy in biological applications [13-19]. The SVM is a maximum margin classifier that can solve non-linear classification problems by learning an optimal separating hyperplane in a higher-dimensional feature space. By use of non-linear kernel functions such as a Gaussian kernel, complex and non-linear decision functions can be learned by the SVM. LS-SVM is a reformulation to standard SVM. It is closely related to regularization networks and Gaussian processes but it additionally emphasizes and exploits primal-dual interpretations from optimization theory. In our experiment, LS-SVM mapped the original input space into a high dimension feature space by a Gaussian kernel and then learns a smoothest hyperplane to fit the training data. From statistical learning theory, it can be expected that this hyperplane would have excellent generalization ability and has minor local extreme value. Together, UD has the ability to greatly reduce the number of experiments while not to alter the representativeness and LS-SVM would yield better generalization for regression problems on finite samples. Thereupon, the integrated application of UD and LS-SVM would have high prediction accuracy and would contribute to transfection optimization.

## Conclusion

This paper investigates the integrated application of UD and LS-SVM to transfection, for obtaining precise information on the optimal conditions. Based on our experiments, UD and LS-SVM appear to have high efficiency and perform well even when undergone experiments are extremely scarce. With the established model, we are able to gain the optimal transfection conditions and the highest transfection efficiency that can be reached. Thus, the required time and experiments to improve transfection efficiency can be greatly reduced while the achieved efficiency may even be higher than traditional methods. It seems that LS-SVM has higher accuracy in the prediction of optimal transfection conditions than it does in the prediction of highest transfection efficiency; nevertheless, we usually have higher stringency of the information on optimal transfection conditions. It should be pointed out that the vigor of host cells and the purity of plasmid have crucial effect on the transfection efficiency too. However, these factors are uncontrollable in most settings. Further interpretation of the results obtained from other host cell lines is required. These issues are part of our ongoing research.

## Methods

### Cell Culture

The 293FT cell line was maintained in DMEM supplemented with 100 mL/L fetal calf serum, 2 mmol/mL L-glutamine, 100 *μ*g/mL penicillin and 100 units/mL streptomycin. The cells were incubated in a humidified incubator at 37°C containing 50 mL/L CO_2_. Cell viability was estimated by the trypan blue dye exclusion method. The 293FT cells were seeded into 24-well plates 24 h prior to transfection. Three wells of cells were transfected for every experiment. The cells were transfected using LipofectAMINE 2000 (abbreviated as LipofectAMINE) cationic liposome (Invitrogen, Carlsbad, California, USA) and the cells were harvested 36 h after transfection. Transfection efficiency was evaluated by calculating the ratio of cells that express green fluorescent protein (GFP) by using flow cytometer (COULTER EPICS XL, Beckman, USA). The experiments were performed in duplicate.

### Uniform Design

On the basis of orthogonal design, UD as a new experimental design method was proposed by Fang *et al *in 1980s. The characteristics of UD are taking no account of regular comparability, completely ensuring the uniformity, and distributing the test points in the experimental scope adequately and uniformly. UD finds good representative points uniformly scattered over the sample space for a much more efficient parameter search. It is one kind of space filling designs that can be used for computer techniques. Suppose there are s samples of interest over a domain *C*^*S*^. The goal here is to choose a set of m points *p*_*m *_= {*θ*_1_, ..., *θ*_*m*_} ⊂ *C*^*S *^such that these points are uniformly scattered on *C*^*S*^.

### Experimentations

In a protocol consists of 15 experiments, amount of liposome, plasmid, and the number of seeded cells were set as independent variables while transfection efficiency was set as dependent variable. Each independent variable had 15 levels. The ranges of independent variables were set according to the instruction of manufacturer. The protocol was performed according to the principle of UD (Table 1). Each transfection efficiency (dependent variable) was calculated by flow cytometer. The expression of GFP in each experiment was also observed by fluorescence microscope (Eclipse 80I, Nikon, Tokyo, Japan). A model was constructed by using LS-SVM. The respective fitted value to each measured transfection efficiency was also deduced from the established model. Another protocol consisting of 10 experiments was designed centering on the predicted optimal conditions at which the dependent variable would reach the maximum (Table 2). And the observed GFP expression was shown in Figure 1 and Figure 2. All the observed data in Table 1 and Table 2 were the mean values of three independent experiments.

### Development of the LS-SVM based models for prediction of transfection efficiency

In regression formulation, the goal is to estimate an unknown continuous-valued function based on a finite number set of noisy samples (**x**_*i*_, *y*_*i*_), (*i *= 1, ..., *n*), where d-dimensional input is **x **∈ *R*^*d *^and the output is *y *∈ *R*. In SVM regression formulations, the input X is first mapped into a m-dimensional feature space using some fixed (nonlinear) mapping, and then a linear model is constructed in this feature space [8]. Using mathematical notation, the linear model (in the feature space) *f*(**x**, *ω*) is given by Equation (1), where *g*_*j *_(**x**), *j *= 1, ..., *m *denotes a set of nonlinear transformations, and b is the "bias" term.

(1)

The quality of estimation is measured by the loss function *L*(*y*, *f*(**x**, *ω*)). SVM regression uses a new type of loss function called *ε*-insensitive loss function proposed by Vapnik [6]:

(2)

SVM regression tries to reduce model complexity by minimizing ||*ω*||^2^. In addition, it introduces (non-negative) slack variables *ξ*_*i*_, *i *= 1, ... *n *to measure the deviation of training samples outside the *ε*-insensitive zone. Thus, SVM regression is formulated as minimization of the following function:

(3)

Compared with simple SVM, LS-SVM computes the solution by solving a linear system instead of quadratic programming. This is due to the use of equality instead of inequality constraints in the above problem formulation. It is well known that LS-SVM generalization performance (estimation accuracy) depends on a good setting of meta-parameters parameters C and the kernel parameters. The main performance metric of LS-SVM is the prediction risk (Equation (4)), defined as mean square error (MSE), between estimated values derived from LS-SVM and true values for testing inputs.

(4)

Therefore, for ensuring good generalization performance, the main issue on LS-SVM application depends on the proper setting of these parameters for a given data set. Selecting a particular kernel type and kernel function parameters is usually based on application-domain knowledge and should also reflect distribution of inputted values of the training data [20]. Here, we showed example of SVM regression using radial basis function (RBF) kernels (Equation (5)), where the RBF width parameter *γ *should reflect the distribution/range of x-values of the training data.

(5)

The LS-SVM based model building processes were carried out by using the software package named LS-SVMlab1.5 available at . This toolbox provides in-depth functionality of SVM. Functions include tuning, optimizing, validating, and training SVMs. Significantly, it provides a good visual representation of the trained LS-SVMlab. The meta-parameters of the LS-SVM model with a Gaussian kernel function are *γ *(the width of the Gaussian kernels) and C (regularization factor). The model construction process consisted consecutively of: i) selection of the inputted variables, ii) selection of the training parameters (C and *γ*), iii) construction of the model, iv) performance evaluation by validation data.

### Contribution analysis of independent variables

The contribution of specific variable was evaluated by using a method based on LS-SVM. Random alteration of the value of a specific variable has a similar effect to take out of this variable. However, the feature space keeps unchanged when it undergoes this process. There are only three variables, so that we can analyze them one by one. The analysis process was described as follows: i) select a variable, such as the density of cells; ii) random exchange the 15 training samples value of cell density and keep other two variables (the amount of plasmid and the amount of LipofectAMINE) unchanged; iii) train another LS-SVM using the same parameters (*γ *= 42, C = 1500); iv) test the trained LS-SVM on 10 testing samples, get the predicated value and MSE; v) repeat step ii to step iv for 10 times, get the average predicated value and average MSE; vi) repeat step i to step v on other two variables. The variable with the biggest MSE has the most important effect on the depend variable.

## Authors' contributions

JSP and MZH generated the idea. JSP, MZH and JLR designed the study. JSP, MZH, JYC, JD and HXS performed the experiments. QFZ and HZW performed the analysis of the transfection data and generated the model. LKL and DQY tested the model. JSP, MZH, QFZ and HZW wrote the draft manuscript. JSP, MZH and JLR proofread the manuscript. All authors participated in production of the final version of the manuscript, read it and approved it.
